# Effect of acidified milk feeding on the intake, average daily gain and fecal microbiological diversity of Holsten dairy calves

**DOI:** 10.5713/ajas.19.0412

**Published:** 2019-10-22

**Authors:** Yong Chen, Yan Gao, Shuxin Yin, Shuai Zhang, Lu Wang, Yongli Qu

**Affiliations:** 1 Heilongjiang Provincial Key Laboratory of Efficient Utilization of Feed Resources and Nutrition Manipulation in Cold Region, College of Animal Science and Veterinary Medicine, Heilongjiang Bayi Agricultural University, Daqing 163319, China

**Keywords:** Calf, Acidified Milk, Feed Intake, Average Daily Gain, Fecal Microbial Diversity

## Abstract

**Objective:**

To evaluate the effect of feeding acidified milk on the growth and fecal microbial diversity of dairy calves.

**Methods:**

Twenty healthy 3-day-old female Holstein calves with similar body weights were selected and randomly divided into two groups. One group was fed pasteurized milk (PM, Control), while the other was fed acidified milk (AM) *ad libitum* until weaned (day 60). The experiment lasted until day 180.

**Results:**

There was no difference in the nutritional components between PM and AM. The numbers of *Escherichia coli* and total bacteria in AM were lower than in PM. At 31 to 40 and 41 to 50 days of age, the milk intake of calves fed AM was higher than that of calves fed PM (p<0.05), and the solid feed intake of calves fed AM was higher than that of calves fed PM at 61 to 90 days (p<0.05). The average daily gain of calves fed AM was also higher than that of calves fed PM at 31 to 60, 61 to 180, and 7 to 180 days (p<0.05). The calves fed AM tended to have a lower diarrhea rate than those fed PM (p = 0.059). *Bacteroides* had the highest abundance in the feces of calves fed AM on day 50, while *Ruminococcaceae_UCG_005* had the highest abundance in the feces of calves fed AM on day 90 and calves fed PM on days 50 and 90. At the taxonomic level, the linear discriminant analysis scores of 27 microorganisms in the feces of calves fed AM and PM on days 50 and 90 were higher than 4.0.

**Conclusion:**

Feeding AM increased calf average daily gain and affected fecal bacterial diversity.

## INTRODUCTION

Milk is a suitable food for bacteria to proliferate. The total number of bacteria increased from 1×10^5^ colony forming units (cfu)/mL to approximately 1.8×10^7^ cfu/mL after 24 h of storage at approximately 23°C [1]. The low pH value of milk or milk replacer (MR) can slow the growth of bacteria, so neither milk nor MR requires cold storage for short periods and can be kept in feeders for calves for longer periods (up to 3 d) [2]. Recently, dairy farms have renewed interest in feeding acidified milk (AM) or MR for *ad libitum* consumption to save laborers in North America and other countries. Todd et al [3] reviewed a suitable target pH range of AM that was between 4.0 and 4.5.

Some studies showed that the feed intake and average daily gain (ADG) of calves fed acidified MR was not different from that of calves fed normal MR when there was no difference in intake and milk composition [4–6]. Woodford [7] reported that feeding calves acidified MR *ad libitum* did not improve the digestion and absorption of nutrients. Jaster et al [8] pointed out that the fecal score of calves fed AM was normal. Other studies showed that calves fed AM or MR had a higher frequency of feeding [2,5], intake [9], and weight gain [10].

Feeding acidified MRs prevented the rapid growth of pathogenic organisms in the alimentary tract [2,11]. Therefore, some results showed that feeding acid milk or MR improved the fecal consistency score [8], decreased the proportion of diarrhea days [12] and reduced the diarrhea rate [13].

Calves tend to be fed in free stalls in large dairy farms in Heilongjiang Province, China. Heilongjiang Province is located in the farthest north and highest latitude of China and has low temperatures, even in summer. This condition is suitable for the use of AM or MR. This study mainly explored the effects of free-access AM on calf growth performance and fecal microorganisms compared with pasteurized milk (PM).

## MATERIALS AND METHODS

### Animals and experimental design

All procedures were approved by the Animal Care and Use Committee of Heilongjiang Bayi Agricultural University. The study was conducted on a large commercial dairy farm (Anda City, Heilongjiang Province, China). Twenty healthy 3-day-old female Holstein calves with similar body weights were selected and randomly divided into two groups; each calf was kept in a pen (2.3 m×1.5 m). One group was fed PM (control), and the other was fed AM *ad libitum* until weaned. The PM was bunk tank milk pasteurized by being heated to 63°C to 65°C for 30 min, and the AM was bunk tank milk acidified by formic acid (35 mL 8.5% formic acid was added to 1,000 mL milk and intermittently stirred at 4°C in a fully automatic stirring refrigeration tank for 10 h to ensure the pH value was between 4.0 and 4.5). The milk was fed to the calves using a 2-L milk can with a nipple, and it was cleaned every three days.

All calves had free access to the same commercial pellet starter (crude protein, 20% of dry matter [DM]; metabolizable energy, 14 MJ/kg DM) before weaning. After weaning, all of them were fed on 1.8 kg commercial pellet per day (the feeding amount was adjusted every two weeks according to calf weight gain: 35 to 45 g/kg/d) and had free access to alfalfa and Chinese wild rye. The calves had *ad libitum* access to water during the experiment.

### Sampling and analysis of milk

The PM and AM were sampled every 15 days before being fed to the calves. Half of the sample—approximately 50 mL —was transferred to a dairy herd improvement bottle with 5% potassium dichromate at 4°C and analyzed for milk composition (lactose, fat, protein, total solids and urea) by Foss Milkoscan 4000 (Foss Electric, Hillerød, Denmark). The other half was stored in a sterile centrifugal tube at 4°C to determine the bacterial count within 2 h after collection.

### Bacterial plate count

The milk samples were diluted by 10-fold to 10^−7^, and 1 mL of every diluted gradient of milk was added to sterile medium to determine the concentration of bacteria after culture at 37°C for 48 h in an incubator. Violet red bile agar medium was used for *Escherichia coli* (*E. coli*) counts after culturing at 37°C for 24 h in an incubator. *Lactobacillus* agar medium was used for *Lactobacillus* count after culture at 37°C for 24 h in an incubator.

### Intake, body weight, average daily gain, and diarrhea rate of calves

The feeding amount and leftover amounts of milk and solid feed were recorded every day to calculate the daily intake of milk and solid feed during the experiment. Calves were weighed before morning feedings at 7, 30, 60, 90, and 180 days of age to calculate the ADG. The calf feces was observed daily until the age of 60 days (weaning) and was identified as diarrhea if it turned out to be paste-like or more liquid. The diarrhea rate (%) was the number of diarrhea days of all calves per treatment / (calf number per treatment × total performed days of fecal scoring) × 100%.

### Analysis of microbial diversity in the feces of calves

Five calves with similar weights were randomly selected from each treatment for collecting fecal samples before the morning feeding on days 50 and 90 (A50, fecal samples of calves fed AM on day 50; A90, fecal samples of calves fed AM on day 90; P50, fecal samples of calves fed PM on day 50; P90, fecal samples of calves fed PM on day 90). The samples were scraped with sterile gloves from the rectal end, immediately placed in liquid nitrogen, and frozen in a freezer at −80°C using sterile cryopreservation tubes.

DNA was extracted by the cetyltrimethylammonium bromide method [14]. DNA concentration and purity were visually monitored on 1% agarose gels. The DNA was diluted to 1 ng/μL using sterile water. The DNA was amplified using the barcode-specific primer set (341F: 5′ - CCTACGGGRB GCASCAG - 3′, 806R: 5′ - GGACTACNNGGGTATCTAAT - 3′), which targets the V3-V4 region of the bacterial 16S rRNA gene. All PCRs were performed using Phusion High-Fidelity PCR Master Mix (New England Biolabs, Beverly, MA, USA) with the following program: 98°C for 1 min, 35 cycles of 98°C for 10 s, 50°C for 3 s and 72°C for 30 s, followed by 72°C for 5 min. The 16S rDNA was sequenced using the Illumina HiSeq sequencing platform. Sequencing services, database construction and statistical analysis were completed by Beijing Nuohe Zhiyuan Science and Technology Co., Ltd. (Beijing, China).

### Sequencing data analysis

All effective tags of samples were clustered using Uparse (version 7.0.1001) [15], and the sequences were clustered into operational taxonomic units (OTUs) by 97% identity. At the same time, the most frequent sequences in OTUs were selected as the representative sequences of OTUs. The representative OTUs were annotated and analyzed using the SSURRNA database of SILVA (http://www.arb-silva.de/) (threshold value: 0.8–1) by Mothur [16,17] to obtain and classify at various levels: phylum, class, order, family and genus. Muscle [18] (version 3.8.31, http://www.drive5.com/muscle/) was used for multiple sequence alignment quickly to gain the phylogenetic relationships of all represented OTUs. The OTU-level alpha diversity of the bacterial communities was determined using various diversity indices and calculated using procedures within QIIME (version 2).

### Statistical analysis

The main effects of milk feeding treatment (PM and AM) on milk composition, milk bacteria count, calf feed intake, body weight and ADG were analyzed using the Proc T TEST procedure of SAS 9.0 (SAS Institute, 2002), and the least-square-means with SE are presented. The diarrhea incidence rates data were compared using the χ^2^ test. A probability (p) value of <0.05 was considered statistically significant, whereas differences were considered to show a statistical trend when 0.05<p<0.10. Linear discriminant analysis effect size (LEfSe) was used to further compare the relative abundances of microbial taxa of the feces of calves fed AM and PM on days 50 and 90, and R software was used for the permutation test [19]. Significant differences were considered by a linear discriminant analysis (LDA) score >4 and p<0.05.

## RESULTS

### Differences in milk composition and bacterial count

The contents of lactose, fat, protein and total solids in PM and AM were not different, but the quantity of milk urea content in AM was higher than that in PM (p<0.001) (Table 1). The AM had a lower total bacterial count (p<0.001) and *E. coli* count (p = 0.015) than PM had; however, there was no difference in the *Lactobacillus* count (Table 1).

### Effect of acidified milk on feed intake, body weight, average daily gain, and diarrhea rate of calves

The milk intake of calves fed AM was higher than that of calves fed PM at 31 to 40 days of age (p = 0.004) and 41 to 50 days of age (p<0.001) (Table 2). In addition, the solid feed intake of calves fed AM was also higher than that of calves fed PM at 61 to 90 days of age (p<0.001), while the milk and solid feed intake were not different at other days of age, including the entire experimental period (Table 2).

At 7 and 30 days of age, there was no difference in body weight between the two groups (Table 3). However, the body weight of the AM group was higher than that of the PM group on days 60, 90, and 180 (p<0.05). Moreover, the AM diet also affected the ADG of calves. The ADG of calves in the AM group was higher than that of calves in the PM group on days 31 to 60, 61 to 180, and 7 to 180 (p = 0.021, p = 0.008, p<0.001).

There was no significant difference between the two groups (Table 4), although the diarrhea incidence rate of calves fed AM was lower than that of calves fed PM (p = 0.059).

### Microbial diversity in the feces of calves fed AM and PM

**Figure 1:**
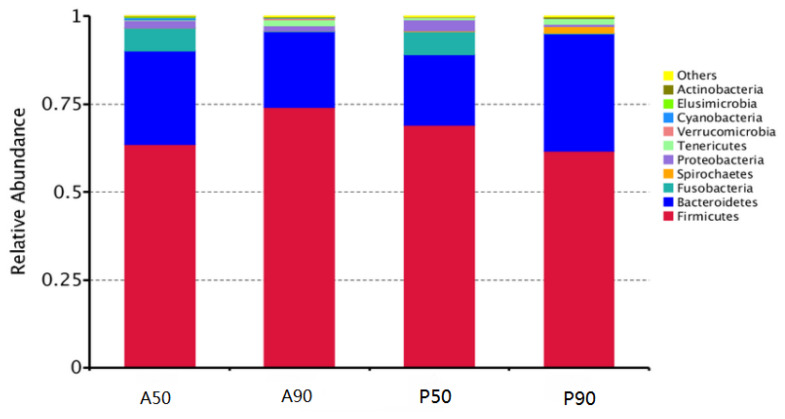
Average relative abundance of microorganisms at the phylum level in the feces of calves fed pasteurized milk (PM) and acidified milk (AM) *ad libitum*. A50, feces samples of calves fed AM on day 50; A90, feces samples of calves fed AM on day 90; P50, feces samples of calves fed PM on day 50; P90, feces samples of calves fed PM on day 90.

**Figure 2:**
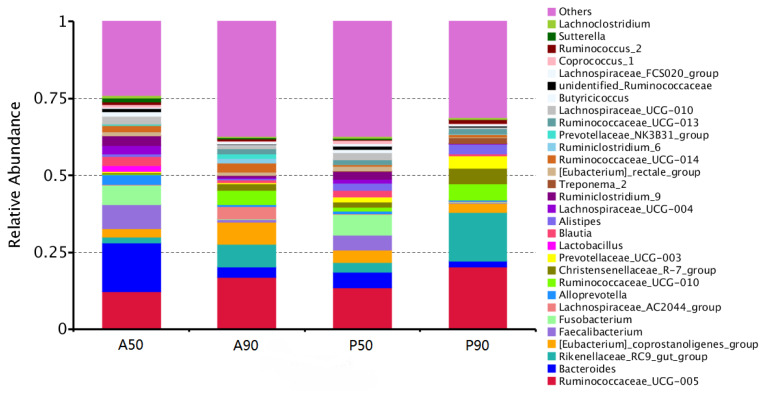
Average relative abundance of microorganisms at the genus level in the feces of calves fed pasteurized milk (PM) and acidified milk (AM) *ad libitum*. A50, feces samples of calves fed AM on day 50; A90, feces samples of calves fed AM on day 90; P50, feces samples of calves fed PM on day 50; P90, feces samples of calves fed PM on day 90.

The bacteria with the highest abundance at the phylum level were *Firmicutes*, followed by *Bacteroidetes* in the feces of all calves fed AM or PM on days 50 and 90 (Figure 1). *Bacteroides* showed the highest abundance in the feces of calves fed AM on day 50 (A50), while *Ruminococcaceae_UCG_005* had the highest abundance in the feces of calves fed AM on day 90 (A90) and PM on days 50 (P50) and 90 (P90) (Figure 2).

**Figure 3:**
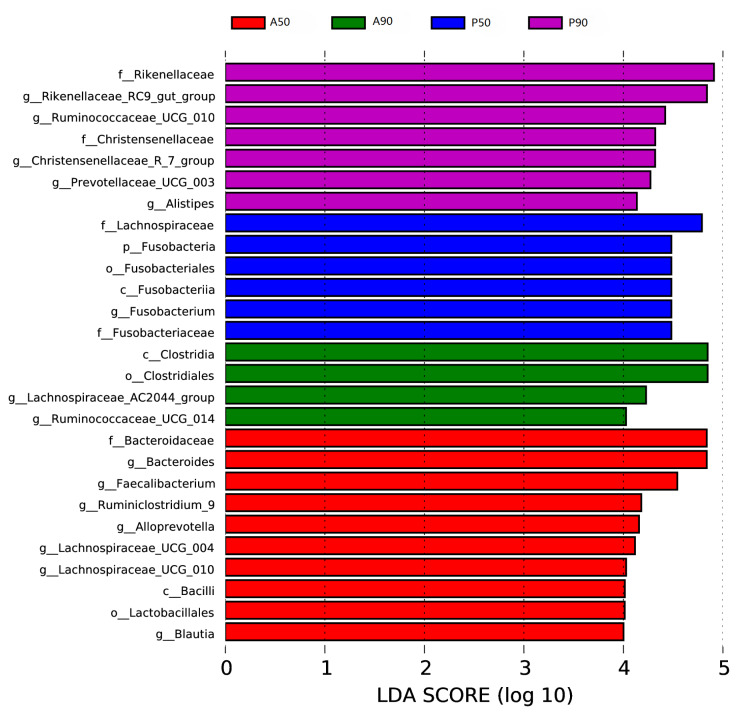
Association of specific microbiota taxa of the microbiota in the feces of calves fed pasteurized milk (PM) and acidified milk (AM) *ad libitum* by linear discriminant analysis (LDA) effect size (LEfSe). Significant differences are defined as p<0.05 and LDA score >4.0. A50, feces samples of calves fed AM on day 50; A90, feces samples of calves fed AM on day 90; P50, feces samples of calves fed PM on day 50; P90, feces samples of calves fed PM on day 90.

The LDA scores of 27 microorganisms in the feces of calves fed AM and PM on days 50 and 90 were higher than 4.0 (Figure 3). *Bacteroidaceae*, *Bacteroidetes*, *Faecalibacterium*, *Ruminiclostridium_9*, *Alloprevotella*, *Lachnospiraceae_UCG_ 004*, *Lachnospiraceae_UCG_010*, *Bacilli*, *Lactobacillales*, and *Blautia* were the dominant bacteria, and *Bacteroidaceae* and *Bacteroidetes* had the highest LDA score (p<0.05) in the fecal microorganisms of calves fed AM on day 50 (A50). *Lachnospiraceae*, *Fusobacteria* (phylum), *Fusobacteria* (class), *Fusobacteriales*, *Fusobacteriaceae*, and *Fusobacterium* were the dominant bacteria, and *Lachnospiraceae* had the highest LDA score (p<0.05) in the fecal microorganisms of calves fed PM on day 50 (P50). *Clostridia*, *Clostridiales*, *Lachnospiraceae AC2044_group*, and *Ruminiclostridium_UCG_014* were the dominant bacteria, and *Clostridia* and *Clostridiales* had the highest LDA score (p<0.05) in the fecal microorganisms of calves fed AM on day 90 (A90). *Rikenellaceae*, *Rikenellaceae_RC9_gut_group*, *Ruminiclostridium_UCG_010*, *Christensenellaceae_R_7_group*, *Prerotellaceae_UCG_003*, and *Alistipes* were the dominant bacteria (p<0.05), and *Rikenellaceae* had the highest LDA score (p<0.05) in the fecal microorganisms of calves fed PM on day 90 (P90).

## DISCUSSION

### Differences in milk composition and bacterial count

Zou et al [20] reported that there was no difference in milk composition—milk fat, milk protein and nonfat solids—between pasteurized abnormal milk and acidified abnormal milk. In this study, the AM had no difference in lactose, milk fat, milk protein and total solids from PM, but the urea content was higher than that in PM. This may be due to the low pH value of AM (pH approximately 4.2). Some studies show that most bacteria in milk can secrete urea-decomposing urease [21]; however, AM (pH near 4.5) inhibited the growth of bacteria [22], leading to the reduction of urease and the increase of urea content in AM.

Microorganisms still survive in milk after intensive heating [23]. Low pH of milk (pH 4.1 to 4.4) had enough bacteriostatic effect, especially for *E. coli* [22]. In the present study, the total counts of bacteria and *E. coli* in AM were lower than in PM, which indicated that the acidification of milk strongly inhibited the growth of bacteria. This result was consistent with the study of Todd et al [11] on acidified MRs.

### Effect of acidified milk on feed intake, body weight, average daily gain, and diarrhea rate of calves

Erickson et al [2] reported that 6-week-old calves fed acidified MR had higher nitrogen retention than the calves fed general MR, but acidified MR was not beneficial to 1-week-old calves. Moreover, the higher frequency of feeding AM or acidified MR increased the feed intake [2,11]. As calves consumed more milk or feed, the body weight of calves fed AM was higher than those fed PM in the current study. AM with low pH provides more suitable pH in the gastrointestinal tract, and the lower pH reduces the rate of infectious scours [10, 24]. Todd et al [11] found that the odds of disease and mortality were not different between the calves fed MR and acidified MR, but acidified MR had less coliform and aerobic bacteria. Our results showed that AM had lower counts of total bacteria and *E. coli* compared with PM. This is beneficial to alimentary tract health, leading to a lower diarrhea rate in calves fed AM in comparison with those fed PM.

### Microbial diversity in the feces of calves fed AM and PM

In this study, the main gut microbe in the feces of all calves at different times was *Firmicutes*, followed by *Bacteroidetes*, which is consistent with the results of other studies [25–28]. The AM diet did not change the phylum level microbiota.

Deng et al [28] showed that an acidified waste milk diet produced similar bacterial diversity in colon mucosa and digesta samples and rectum feces in calves, with present results at the generic level. The abundance of *Bacteroidetes* in the feces of calves fed AM before weaning positively correlated with the colonization resistance of gut harmful microorganisms, which would prevent the entry of pathogens in the early stage of infection [25,29]. The results of Todd et al [5,11] showed that an AM replacer diet decreased harmful bacteria and had a decreasing trend of morbidity and mortality, which were beneficial to calf growth. Heyman and Ménard [30] pointed out that feeding acidified formula powdered milk to infants changed intestinal pathological conditions and decreased the incidence of diarrhea. Moreover, Burton et al [31] reported that AM reduced postprandial inflammation.

Yin et al [32] did not find a difference in the bacterial relative amount in the mouse gut after stopping the AM diet for several days compared with normal milk feeding. In the present study, the fecal microbes of calves fed AM or PM also had similar relative abundance after weaning, which is likely the reason for the same diet and living conditions.

According to some results on intestinal microbes [33–35], the feces of calves fed AM before weaning had many beneficial bacteria for the gut, which would improve gut health and low occurrence of diarrhea [2,10]. After weaning, although the feces of calves fed AM and PM had different bacterial taxa, *Ruminococcaceae_UCG_005* had the highest abundance in feces of calves fed AM and PM, which would improve the fiber utilization ability of calves [36].

## CONCLUSION

Compared with PM, AM inhibited the growth of bacteria, especially *E. coli*, in milk. Feeding AM can increase calf feed intake and improve average daily gain. Feeding AM did not change the main fecal bacterial relative abundance at the phylum level; however, it affected the genus relative abundance. Moreover, 27 microbe generic biomarkers in the feces of all calves on days 50 and 90 showed significance.

## Figures and Tables

**Table 1:** The composition and bacteria count of the pasteurized and acidified milk

Items	PM	AM	SEM	p-value
Lactose (%)	4.20	4.29	0.05	0.194
Milk fat (%)	4.44	4.63	0.07	0.125
Milk protein (%)	3.73	3.55	0.08	0.221
Urea (mg/dL)	17.63	59.32	0.73	<0.001
Total solids (%)	13.06	13.78	0.29	0.870
Total bacterial count (×10^3^ cfu/mL)	146.69	93.33	5.42	<0.001
*Lactobacillus* count (×10^2^ cfu/mL)	76.75	82.88	5.79	0.413
*E. coli* count (cfu/mL)	35.77	19.96	4.85	0.015

PM, pasteurized milk; AM, acidified milk; SEM, standard error of the mean.

**Table 2:** The daily intake of milk and starter of calves fed pasteurized and acidified milk *ad libitum*

Items	Days of age (d)	PM	AM	SEM	p-value
Milk (L)	4–20	6.28	6.43	0.24	0.661
	21–30	8.80	8.99	0.14	0.331
	31–40	11.38	13.08	0.44	0.004
	41–50	12.09	14.18	0.02	<0.001
	51–60	7.39	7.40	0.52	0.995
	4–60	9.08	9.51	0.37	0.228
Solid feed (kg)	4–30	0.03	0.03	0.01	0.860
	31–60	0.46	0.51	0.11	0.710
	61–90	2.04	2.30	0.08	<0.001
	91–180	4.58	4.79	0.24	0.475
	4–180	2.71	2.90	0.13	0.117

PM, pasteurized milk; AM, acidified milk; SEM, standard error of the mean.

**Table 3:** Performance of calves fed pasteurized and acidified milk *ad libitum* (kg)

Items	Days of age (d)	PM	AM	SEM	p-value
Body weight	7	44.40	44.60	1.44	0.911
	30	65.60	67.60	1.47	0.535
	60	80.33	96.78	2.20	<0.001
	90	113.00	130.22	5.85	0.022
	180	214.67	267.22	9.31	<0.001
Average daily gain	7–30	0.77	0.84	0.07	0.505
	31–60	0.79	1.05	0.05	0.021
	61–180	1.15	1.44	0.07	0.008
	7–180	0.97	1.28	0.06	<0.001

PM, pasteurized milk; AM, acidified milk; SEM, standard error of the mean.

**Table 4:** The diarrhea rate of calves fed pasteurized and acidified milk *ad libitum* (%)

Days	PM	AM	p-value
3–10	13.89	5.56	0.059
11–30	10.00	5.56	0.297
31–60	9.63	5.19	0.179

PM, pasteurized milk; AM, acidified milk.
